# Standardization and application of a modified RFLP-PCR methodology for analysis of polymorphisms linked to treatment resistance in *Ancylostoma braziliense*

**DOI:** 10.1186/s13071-018-3125-9

**Published:** 2018-10-09

**Authors:** Luis Fernando Viana Furtado, João Guilherme Scarpelli Magalhães, Élida Mara Leite Rabelo

**Affiliations:** Departamento de Parasitologia, L4 237, Laboratório de Parasitologia Molecular, Universidade Federal de Minas Gerais, Instituto de Ciências Biológicas, Avenida Presidente Antônio Carlos, 6627, Pampulha, Belo Horizonte, Minas Gerais CEP 31270-901 Brazil

**Keywords:** Hookworm, *Ancylostoma braziliense*, Resistance, Beta-tubulin

## Abstract

**Background:**

Single nucleotide polymorphisms (SNPs) in codons 167, 198 and 200 of the beta-tubulin isotype 1 gene are associated with benzimidazoles resistance in many helminths. Codon 167 mutation has never been described in hookworms; however, polymorphisms in codons 198 and 200 have been described for *Ancylostoma caninum* and *Necator americanus*. These mutations have never been investigated in *Ancylostoma braziliense*; therefore, it is not known if they are present in this species and whether they are correlated with treatment resistance. The RFLP-PCR technique has been used to analyze these polymorphisms in some nematodes, but depending on the species, these alterations do not create or eliminate any restriction enzyme cleavage site, making it impossible to use this technique. Here, we describe the standardization and application of a modified RFLP-PCR technique for detecting polymorphisms in individual *A. braziliense* worms recovered from naturally infected dogs in two Brazilian states.

**Results:**

The molecular techniques used were sensitive, specific, and easy to apply. To our knowledge, we report for the first time the presence of a polymorphism at codon 198 of the beta-tubulin gene of *A. braziliense* (1/81; 95% CI: 0–3.69%).

**Conclusions:**

It is not known whether the presence of the mutation in codon 198 of the beta-tubulin gene of *A. braziliense* has importance for this parasite. However, based on studies of other helminths, it is possible that this polymorphism is directly related to the resistance to benzimidazoles. This may be a major concern, since this nematode has considerable relevance as a parasite of canids and felids and as one of the agents of cutaneous larva migrans in humans. Standardized methodologies will be useful for screening for polymorphisms in the beta-tubulin gene of canine hookworms in a broader population. The method could also be adapted for the analysis of other SNPs in other nematode species.

**Electronic supplementary material:**

The online version of this article (10.1186/s13071-018-3125-9) contains supplementary material, which is available to authorized users.

## Background

*Ancylostoma braziliense* is an intestinal nematode of dogs, cats and some wild animals, and can cause severe damage, such as anemia, to its hosts [1]. This parasite has considerable zoonotic importance as an agent of erythematous and pruriginous inflammation in the dermis, known as cutaneous larva migrans [2].

The periodic treatment of helminths with benzimidazoles can select for treatment-resistant parasites [3]. Single nucleotide polymorphisms (SNPs) at codons 167 (TTC/Phe→TAC/Tyr), 198 (GAG/Glu→GCG/Ala) and 200 (TTC/Phe→TAC/Tyr) in the beta-tubulin isotype 1 gene have been linked to benzimidazole resistance in some helminths [3–5]. Codon 167 mutation has never been described for hookworms [6]. Conversely, SNPs have been described in codon 200 for *Ancylostoma caninum* [7] and in codons 198 [8] and 200 [9] for *Necator americanus*. These mutations have never been investigated in *A. braziliense*; therefore, it is not known if they are present in this species.

Some molecular methodologies have been described in the literature for genotyping SNPs in hookworms, such as ARMS-PCR [7, 10], Tetraprimer ARMS-PCR [11], real-time PCR (qPCR) [12] and Smart Amplification Process 2 (SmartAmp2) [8]. Restriction fragment length polymorphism-polymerase chain reaction (RFLP-PCR) has been used successfully to detect SNPs linked to resistance, and it is a sensitive, specific, low cost and easy to apply technique. Moreover, the process of restriction enzyme digestion yields a quick result by gel analysis. According to Ota et al. [13], this technique is simple, sensitive, and reliable and requires minimal investment in instrumentation; however, it has some limitations, such as in cases where the DNA sequences are not recognized by commercial restriction enzymes or harbor many recognition sites for a single enzyme. Here, we standardize a simple and economical approach based on RFLP-PCR and site-directed mutagenesis followed by RFLP-PCR for genotyping SNPs in two Brazilian populations of *A. braziliense*. We did not have information on the dogs’ treatment history or on the possibility of drug resistance. To our knowledge, we describe for the first time the presence of a mutation at codon 198 of the beta-tubulin isotype 1 gene for this species.

## Methods

### Parasite collection and DNA extraction

Worms were recovered from dogs from two Brazilian state capitals, Teresina (Piauí) and Belo Horizonte (Minas Gerais). The dogs were routinely subjected to euthanasia following the approved procedures of the Municipality Health Centers of the respective Brazilian cities by the Zoonoses Control Center (CCZ). Adult worms were collected from 9 dogs from Piauí and 10 dogs from Minas Gerais during necropsy. Species identification was performed by examining the buccal capsule under a stereomicroscope [14]. DNA was extracted employing a technique described by Waldschmidt et al. [15] from 81 individual worms, including 39 worms collected from Piauí (18 males and 21 females) and 42 worms collected from Minas Gerais (15 males and 27 females).

### Primer design

There was no *A. braziliense* beta-tubulin isotype 1 gene sequence available in the databases. Therefore, we tested primers designed for the beta-tubulin gene of *A. caninum* [7] and sequenced the *A. braziliense* regions flanking the codons of interest. The beta-tubulin isotype 1 gene sequence of *A. braziliense* was deposited in GenBank, under accession number MG866017. The primers used in this study were designed using the program Oligo Explorer 1.4™ (Gene link, USA). Additional file 1: Table S1 shows all the primers designed in this study.

### Construction of controls

To standardize molecular analyses, we first synthesized controls for the presence and absence of mutations for all codons, according to Furtado & Rabelo [16]. To construct a wild-type control allele for codon 167 (N167Ab), an initial PCR amplification was performed with the primers *Fa167Ab* and *Ra167Ab* (415 bp) using genomic DNA from *A. braziliense*. Since codons 198 and 200 are close to each other in the genome, it was possible to construct a single unmutated control for both codons using the primers *Fa198/200Ab* and *Ra198/200Ab* (424 bp). Next, nested PCR was performed using the primers *Fc167Ab* and *Rc167Ab* (306 bp, to codon 167) and the primers *Fc198/200Ab* and *Rc198/200Ab* (308 bp, to codons 198 and 200), and the fragments obtained were sequenced to confirm the absence of mutations in both amplified fragments. The fragments were subsequently cloned using the pGEM™-T Easy Vector System (Promega, Madison, USA), transformed into XL1-blue™ cells (Phoneutria, Belo Horizonte, Brazil) and recovered via miniprep (Wizard™ Plus Miniprep DNA Purification System, Promega, USA).

To construct mutated positive controls for codons 167 (M167Ab), 198 (M198Ab) and 200 (M200Ab), site-directed mutagenesis was performed using the Megaprimer-PCR technique. Cloned N167Ab was used as a template for PCR amplification of codon 167 using the primers *Fc167Ab* and *Rm167Ab* (176 bp), while cloned N198/200Ab was used as a template for PCR amplification of codon 198 and 200 using the primer combinations *Fc198/200Ab* and *Rm198Ab* (132 bp) and *Fc198/200Ab* and *Rm200Ab* (138 bp), respectively. The *Rm167Ab*, *Rm198Ab* and *Rm200Ab* primers were designed to include a mismatch at position 8 of the 5′ end of the primer (see Additional file 1: Table S1) to mimic the mutated sequence. The reaction products were subjected to electrophoresis on 1.0% agarose gels (w/v) (Midsci, Valley Park, USA) with 0.5× TAE buffer, and the gels were stained with GelRed™ (Biotium, Fremont, USA). The fragments were gel excised and purified (Illustra GFX™ PCR DNA and Gel Band Purification Kit, GE Healthcare, Chicago, USA), and their concentrations were determined. Approximately 20 ng of the first reaction product was used as a forward mega-primer in the second reaction in combination with the reverse *Rc167Ab* (306 bp, to codon 167) and *Rc198/200Ab* (308 bp to codon 198 and 200) primers. The products of the three codons were purified, sequenced, cloned and recovered in the same way as described for the negative controls. All PCR amplifications were performed using GoTaq Green Master Mix (Promega), with a final concentration of 0.2 μM for each primer. All amplification steps were performed in a Mastercycler (Eppendorf, Hamburg, Germany) thermocycler according to the following program: 94 °C for 5 min; 30 cycles of 94 °C for 1 min, 57 °C for 1 min, and 72 °C for 1 min; and 72 °C for 8 min. In all amplification runs, a “blank” sample was included in which the DNA was replaced with water.

### Analysis of codons

For the analysis of SNPs in codons 167, 198 and 200 of the beta-tubulin gene of *A. braziliense*, the RFLP-PCR technique was performed. Sequences were analyzed using the NEBcutter V2.0 tool (http://www.labtools.us/nebcutter-v2-0/). The mutation at codon 167 created a restriction site for the enzyme *Rsa*I. Therefore, this enzyme was used to evaluate this codon by RFLP-PCR. However, no restriction sites were found that could differentiate mutated from unmutated sequences for codons 198 and 200. Thus, to analyze codons 198 and 200, site-directed mutagenesis was required to create a site for the restriction enzymes *Dde*I and *Rsa*I, respectively, to differentiate the genotypes by RFLP-PCR.

For codon 167, initial PCR using primers *Fa167Ab* and *Ra167Ab* (415 bp) was performed, followed by nested PCR with the primers *Fc167Ab* and *Rc167Ab* (306 bp). For codons 198 and 200, initial PCR with the primers *Fa198/200Ab* and *Ra198/200Ab* (415 bp) was performed. To introduce the restriction sites, primers for the nested reaction were designed with an alteration at position 3 from the 3′ end for codon 198, which in association with the remaining sequence created a site for the restriction enzyme *Dde*I in the unmutated allele. For codon 200, a mismatch was introduced in the primer *Fsite200Ab* at position 2 from the 3′ end that in combination with the remaining sequence, created an additional restriction site for the enzyme *Rsa*I in the mutated allele. Additional file 2: Figure S1 and Additional file 3: Figure S2 show the methodology used for codons 198 and 200, respectively. Therefore, using the product of this first reaction, nested-PCR was performed for codon 198 with the primers *Fsite198Ab* and *Rc198/200Ab* (214 bp), and another nested PCR was performed for codon 200 with the primers *Fsite200Ab* and *Rc198/200Ab* (208 bp). The three PCR products were subjected to 1.0% (w/v) agarose gel electrophoresis (Midsci) using 0.5× TAE buffer and GelRed® staining (Biotium) to confirm the amplicon sizes. Restriction enzyme digestion of all of the codons was performed with 5 U (codons 167 and 200) or 2 U (codon 198) of each enzyme in individual reactions according to the manufacturer's recommendations. After digestion, the products were subjected to 15.0% (w/v) polyacrylamide gel electrophoresis in 0.5× TBE buffer, followed by GelRed® staining (Biotium). Additional file 4: Table S2 shows the expected fragment sizes after the digestion of each genotype. The confidence interval (CI) for the results was obtained using Graph Prism version 7.0 (GraphPad Software, USA).

## Results

**Fig. 1:**
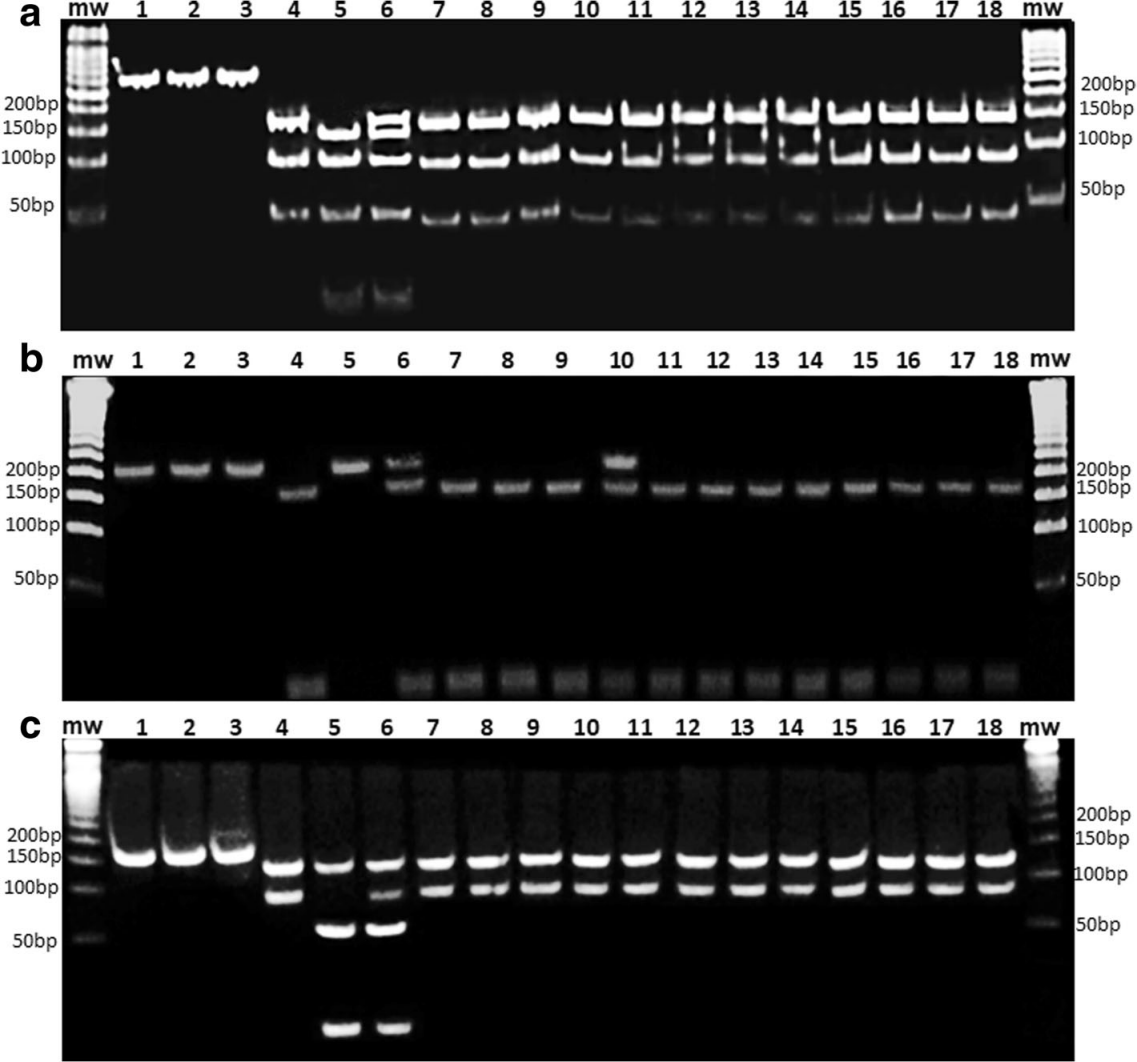
Representative RFLP-PCR results from the analysis of codons 167 (**a**), 198 (**b**) and 200 (**c**) of the *Ancylostoma braziliense* beta-tubulin gene using the restriction *Rsa*I enzyme for codons 167 and 200, and the *Dde*I enzyme for codon 198. Lanes 1–3 contain undigested PCR products from plasmid controls (Lane 1: unmutated plasmid; Lane 2: mutated plasmid; Lane 3: mix of mutated and unmutated plasmid). Lanes 4–6 contain PCR digestion products from control plasmids (Lane 4: unmutated plasmid; Lane 5: mutated plasmid; Lane 6: mix of mutated and unmutated plasmid). Lanes 7–18 contain RFLP-PCR products from *A. braziliense* DNA. Each image is of a polyacrylamide gel (12%) stained with GelRed™ (Biotium, USA). Lane MW: 50 bp molecular weight ladder. Expected fragments sizes (bp): codon 167: unmutated: 162 + 95 + 49, mutated: 138 + 95 + 49 + 24; codon 198: unmutated: 186 + 28, mutated: 214; codon 200: unmutated: 137 + 41, mutated: 137 + 71 + 30

Mutations in codons 167 and 200 in worms from dogs in both states were not found. For codon 198, only one of the specimens from Minas Gerais presented a mutation at codon 198, corresponding to 1.2% (1/81) of the total samples analyzed. This single mutation was present in a heterozygous worm. Figure 1 shows gels demonstrating the mutation screening at codons 167 (1a), 198 (1b) and 200 (1c).

## Discussion

SNPs in the beta-tubulin isotype 1 gene at codons 167 (TTC/Phe→TAC/Tyr), 198 (GAG/Glu→GCG/Ala) and 200 (TTC/Phe→TAC/Tyr) have been linked to benzimidazole resistance in some helminths [3–5]. Earlier detection of these SNPs increases the possibility of intervening to delay the establishment of a drug-resistant worm population. Therefore, there is a need for tests that are sensitive, specific and easy to apply. Here, we describe the standardization and application of a modified RFLP-PCR technique for detecting polymorphisms in individual *A. braziliense* worms from two Brazilian states.

None of the samples evaluated presented a mutation at codon 167. The polymorphism in this codon was first described for *Haemonchus contortus* by Prichard [5], and although this mutation was investigated in dog hookworms [10] and humans [9], it was never described for this group of parasites. In cyathostomes, the mutation at codon 167 has been suggested as the main mechanism of resistance to benzimidazoles [17]. Due to the phylogenetic proximity [18], it was conceivable that this SNP would occur in hookworms; however, this hypothesis was not confirmed by the present study, but the SNP may be found in a larger study or in different geographical localities.

This report is the first description of mutation in codon 198 of the beta-tubulin gene of *A. braziliense* (1/81, 95% CI: 0–3.69). This mutation has been previously investigated in populations of *A. caninum* from Brazil [11] and the USA [12] but has never been reported for this species. According to Silvestre & Cabaret [19], it is possible that this single heterozygous mutation is sufficient to render the worm resistant to benzimidazole treatment, which is concerning. The frequency of this mutation found in the present work can be considered low compared to that found in studies of parasites of livestock [20]. In general, research involving cattle parasites tends to show a high frequency of SNPs in the beta-tubulin isotype 1 gene [21]. We suggest that this fact may be attributed to the control strategies used for different hosts; for example, cattle herds are periodically treated massively with anthelmintics for economic reasons, while this is not usually the case with dogs.

No mutation was observed at codon 200 in any sample analyzed. Rashwan et al. [8] and Schwenkenbecher et al. [22] also did not detect mutation in this codon in *N. americanus*, while Furtado et al. [7] and Diawara et al. [9] detected a mutation in this codon in *A. caninum* and *N. americanus*, respectively. There is no reason to believe that a polymorphism at codon 200 is not present in *A. braziliense*. The history of dogs used in this work is unknown, and there are no reports on the effectiveness of *A. braziliense* treatment in Brazilian populations of dogs or cats. It was not possible to know if these animals had already been treated with an anthelmintic. In fact, this work proposed to carry out a molecular scan for the natural presence of these SNPs in populations of worms isolated from dogs that had not necessarily received previous treatment to identify the potential risk of the large-scale use of this anthelmintic. We suggest that *in vitro* phenotypic tests should be performed to evaluate the sensitivity of hookworm populations in Brazil, which would help to more fully elucidate the resistance and sensitivity of this species to different anthelmintics.

*A. braziliense*, similar to *A. caninum*, is a zoonotic parasite, and although it is not able to develop in humans, it may be responsible for a cutaneous infection called cutaneous larva migrans [2]. Considering that this class of drugs has the same mechanism of action against different developmental stages of the helminth, polymorphisms in the beta-tubulin gene may confer treatment resistance to larvae present in cutaneous infections. Accordingly, there are reports of failure with standard albendazole treatment for this type of infection, highlighting the possibility of treatment resistance [23]. Ideally, to confirm the relationship between failures of benzimidazole treatment for larva migrans and the presence of these SNPs, it would be necessary to perform molecular analyses of resistant larvae directly collected from human skin lesions.

The molecular methodologies standardized here were sensitive and specific for the detection of mutations related to benzimidazole resistance. Since SNPs at codons 198 and 200 of *A. braziliense* do not create or eliminate a cleavage site for any restriction enzyme, it was necessary for the analysis of both codons to introduce a change in one of the PCR primer by site-directed mutagenesis, creating a cleavage site in association with the codon sequence of interest. In this methodology, larger primers for introducing the change in the DNA will better distinguish the genotypes in the gel. However, in the analyses for *A. braziliense*, the intentionally altered primers (*Fsite198Ab* and *Fsite200Ab*) were 30 bp, only slightly larger than the usual primers, although they were able to differentiate the genotypes of interest.

Our group performed analyses of these SNPs in *A. caninum* populations collected from different Brazilian states. These analyses were performed for codons 167 and 200 by ARMS-PCR [7, 10], whereas codon 198 was analyzed by Tetraprimer ARMS-PCR [11]. Although standardized techniques produced reliable results, with high sensitivity and specificity, the standardization was very laborious. The main drawback of these methodologies is that the primers need to strictly pair with the target DNA sequence, without allowing for adjustments in primer pair compatibility. Once the primers pair is not compatible, it becomes impossible to design new primers, rendering these techniques infeasible [16]. However, this type of problem is not observed for the use of RFLP-PCR since, depending on the objective, amplification can be performed using primers that are designed to any sequence that flanks the target region. Furthermore, this method can be easily applied in DNA genotyping of multiple organisms.

Studies based on qPCR and sequencing are described in the literature for the analysis of SNPs in the beta-tubulin isotype 1 gene of helminths [12, 24]. However, these methods, although sensitive, require specific equipment, while RFLP-PCR techniques only require the use of a conventional thermocycler, providing a direct result without the need for graph analysis or chromatograms. Schwenkenbecher et al. [22] detected a mutation in codons 167 and 200 at a very low frequency in *N. americanus* from children who received treatment periodically and associated these data with a possible experimental error in qPCR. Rashwan et al. [8] developed a genotyping assay for SNPs at codons 167, 198 and 200 in *N. americanus* beta-tubulin isotype 1 gene using the SmartAmp2 method. According to these authors, the advantages of this technique are that sophisticated equipment is not needed and only a short time is required for the reaction. On the other hand, the combination of several initiators (five to six) in the same reaction may hinder the proper functioning of these reactions.

## Conclusions

We conclude that a mutation in codon 198 in the beta-tubulin gene of *A. braziliense* exists in the population evaluated, although at a low frequency. The presence of the mutation in this population associated with recurrent treatment can select for resistant strains of parasites, suggesting the need for constant monitoring. This may represent a major problem since this nematode has considerable relevance as a parasite of canids and felids and as an agent of cutaneous larva migrans in humans.

## Additional files

Additional file 1: **Table S1.** Primers used for SNP analysis in this work with their respective annealing temperatures and base substitutions (when applicable). The bases that have been replaced are in bold. (PDF 76 kb)

Additional file 2: **Figure S1.** Representation of the methodology used to analyze codon 198 of the beta-tubulin isotype 1 gene from *Ancylostoma braziliense*. The *Fsite198Ab* primer was designed to add a mutation in the amplicon, regardless of whether there was a mutation at codon 198. The absence of the mutation at codon 198 in combination with the primer-introduced change creates a site for *Dde*I. If the allele is mutated, even the altered primer cannot create a *Dde*I site. Codon 198 is underlined, with the base of interest in bold. (PDF 615 kb)

Additional file 3: **Figure S2.** Representation of the methodology used to analyze codon 200 of the beta-tubulin isotype 1 gene from *Ancylostoma braziliense*. The *Fsite200Ab* primer was designed to add a mutation in the amplicon, regardless of whether there was mutation at codon 200. The presence of the mutation at codon 200 in combination with the primer-introduced change creates a site for *Rsa*I. If the allele does not harbor a mutation, even the altered primer cannot create a *Rsa*I site. Codon 200 is underlined, with the base of interest in bold. (PDF 88 kb)

Additional file 4: **Table S2.** Amplicon sizes after RFLP-PCR for the analysis of codons 167, 198 and 200 of the beta-tubulin isotype 1 gene of *Ancylostoma braziliense*. (PDF 30 kb)

## Abbreviations

ARMS: Amplification-refractory mutation system
PCR: Polymerase chain reaction
RFLP: Restriction fragment length polymorphism
