# Supplementary material for: Standardization and application of a modified RFLP-PCR methodology for analysis of polymorphisms linked to treatment resistance in Ancylostoma braziliense
Source: Parasit Vectors. 2018 Oct 9;11:540. doi: 10.1186/s13071-018-3125-9 (PMC6178248; doi:10.1186/s13071-018-3125-9)
Supplement: Supplementary file 1 — Table S1. Primers used for SNP analysis in this work with their respective annealing temperatures and base substitutions (when applicable). The bases that have been replaced are in bold. (PDF 76 kb) [file 13071_2018_3125_MOESM1_ESM.pdf]

Additional file 1: Table S1. Primers used for SNP analysis in this work with their respective annealing temperatures and base substitutions (when applicable). The bases that have been replaced are in bold.

Códon	Primer (5' – 3')	Change	Annealing temperature (°C)
167	***Fa167Ab***: TGA GCT CGT CGA TAA CGT CC		57
	Fb167Ab: CAG GTA TTT CGC AAC CGT GC		57
	Ra167Ab: TGA GGT CAT CCC CAG TTT GAC		57
	Rb167Ab: AGG AAC ACG ACC AGC GTT T		57
	Rm167Ab: AAC AGA G**T**A CGA GGA CAT AAT C	A → T	57
198/200	***Fa198/200Ab***: TGT TCC TAA AAA GGG GTC GGG		57
	Fb198/200Ab: GCA GTC CAC GTT CCT GCT TA		57
	Fsite198Ab: CTG TGC ACC AAT TGG TCG AGA ACA CAG **C**TG	A → C	60
	Fsite200Ab: ACC AAT TGG TCG AGA ACA CAG ATG AGA C**G**T	G → C	60
	Ra198/200Ab: AAG CGA AGG CAG GTA GTG AC		57
	Rb198/200Ab: ACC GGA CAT TGT TAC AGA CAC T		56
	Rm198Ab: GAA GGT C**G**C ATC TGT GTT CTC	T → G	56
	Rm200Ab: GAT ACA G**T**A GGT CTC ATC TGT	A → T	57
