# Supplementary material for: Standardization and application of a modified RFLP-PCR methodology for analysis of polymorphisms linked to treatment resistance in Ancylostoma braziliense
Source: Parasit Vectors. 2018 Oct 9;11:540. doi: 10.1186/s13071-018-3125-9 (PMC6178248; doi:10.1186/s13071-018-3125-9)
Supplement: Supplementary file 2 — Figure S1. Representation of the methodology used to analyze codon 198 of the beta-tubulin isotype 1 gene from Ancylostoma braziliense. The Fsite198Ab primer was designed to add a mutation in the amplicon, regardless of whether there was a mutation at codon 198. The absence of the mutation at codon 198 in combination with the primer-introduced change creates a site for DdeI. If the allele is mutated, even the altered primer cannot create a DdeI site. Codon 198 is underlined, with the base of interest in bold. (PDF 615 kb) [file 13071_2018_3125_MOESM2_ESM.pdf]

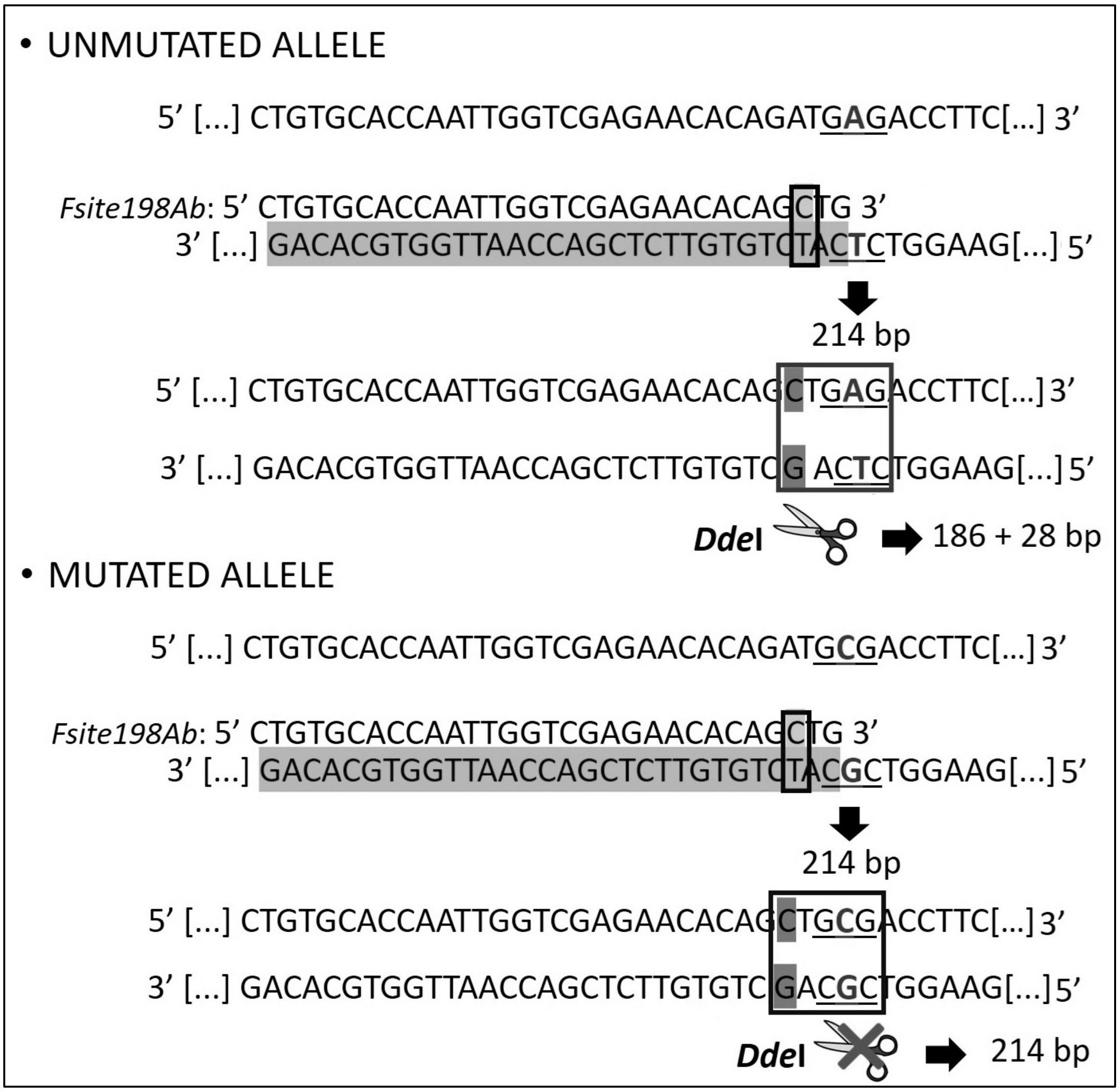

Additional file 2: Figure S1. Representation of the methodology used to analyze codon 198 of the beta-tubulin isotype 1 gene from *Ancylostoma braziliense*. The *Fsite198Ab* primer was designed to add a mutation in the amplicon, regardless of whether there was a mutation at codon 198. The absence of the mutation at codon 198 in combination with the primer-introduced change creates a site for *Dde*I. If the allele is mutated, even the altered primer cannot create a *Dde*I site. Codon 198 is underlined, with the base of interest in bold.
