# Supplementary material for: Standardization and application of a modified RFLP-PCR methodology for analysis of polymorphisms linked to treatment resistance in Ancylostoma braziliense
Source: Parasit Vectors. 2018 Oct 9;11:540. doi: 10.1186/s13071-018-3125-9 (PMC6178248; doi:10.1186/s13071-018-3125-9)
Supplement: Supplementary file 3 — Figure S2. Representation of the methodology used to analyze codon 200 of the beta-tubulin isotype 1 gene from Ancylostoma braziliense. The Fsite200Ab primer was designed to add a mutation in the amplicon, regardless of whether there was mutation at codon 200. The presence of the mutation at codon 200 in combination with the primer-introduced change creates a site for RsaI. If the allele does not harbor a mutation, even the altered primer cannot create a RsaI site. Codon 200 is underlined, with the base of interest in bold. (PDF 88 kb) [file 13071_2018_3125_MOESM3_ESM.pdf]

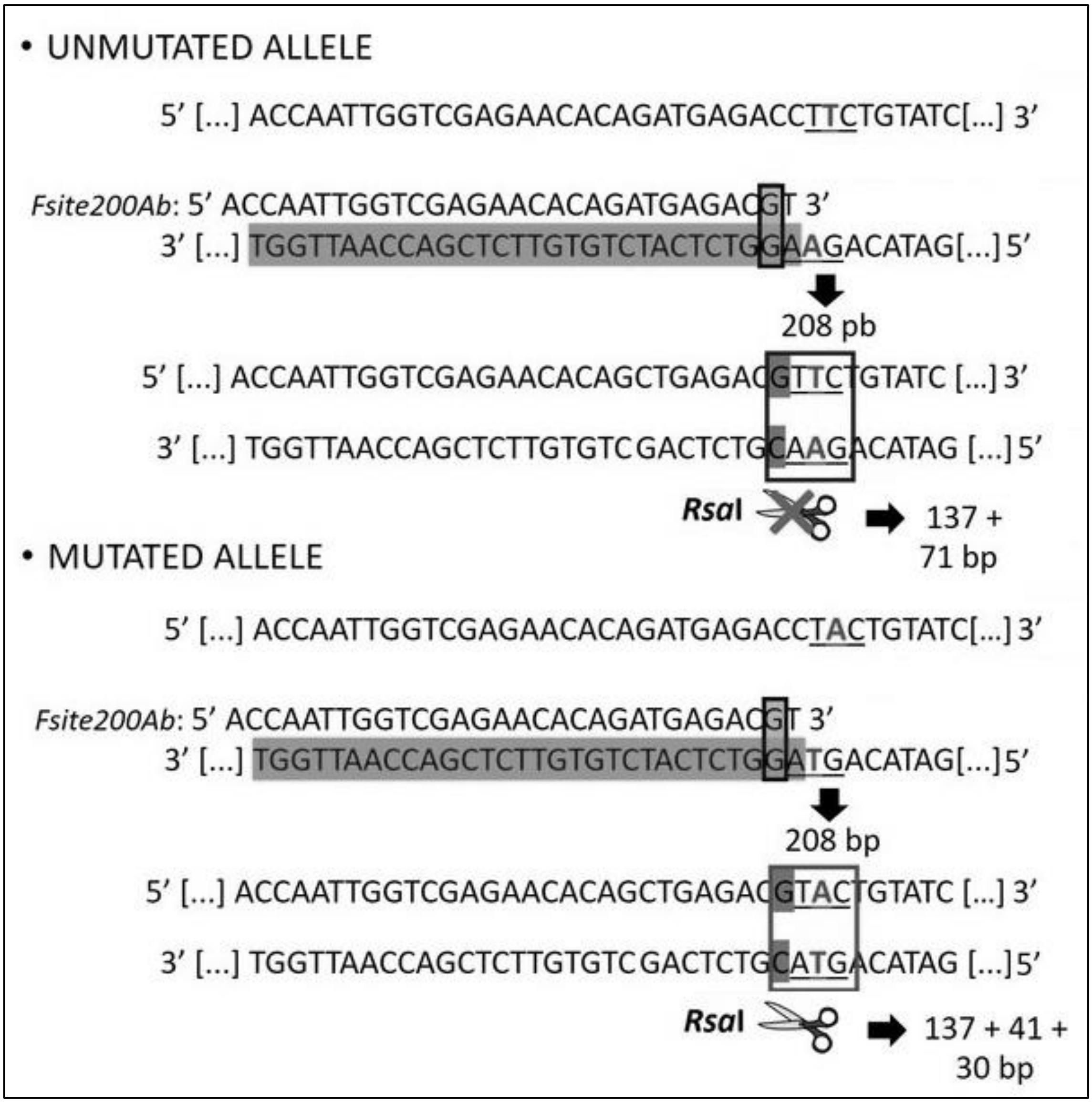

Additional file 3: Figure S2. Representation of the methodology used to analyze codon 200 of the beta-tubulin isotype 1 gene from *Ancylostoma braziliense*. The *Fsite200Ab* primer was designed to add a mutation in the amplicon, regardless of whether there was mutation at codon 200. The presence of the mutation at codon 200 in combination with the primer-introduced change creates a site for *Rsa*I. If the allele does not harbor a mutation, even the altered primer cannot create a *Rsa*I site. Codon 200 is underlined, with the base of interest in bold.
