# Supplementary material for: Standardization and application of a modified RFLP-PCR methodology for analysis of polymorphisms linked to treatment resistance in Ancylostoma braziliense
Source: Parasit Vectors. 2018 Oct 9;11:540. doi: 10.1186/s13071-018-3125-9 (PMC6178248; doi:10.1186/s13071-018-3125-9)
Supplement: Supplementary file 4 — Table S2. Amplicon sizes after RFLP-PCR for the analysis of codons 167, 198 and 200 of the beta-tubulin isotype 1 gene of Ancylostoma braziliense. (PDF 30 kb) [file 13071_2018_3125_MOESM4_ESM.pdf]

Additional file 4: Table S2. Amplicon sizes after RFLP-PCR for the analysis of codons 167, 198 and 200 of the beta-tubulin isotype 1 gene of *Ancylostoma braziliense*.

**Codon**	**Homozygous unmutated**	**Homozygous mutated**	**Heterozygous**
**167**	162 + 95 + 49	138 + 95 + 49 + 24	162 + 138 + 95 + 49 + 24
**198**	186 + 28	214	214 + 186 + 28
**200**	137 + 71	137 + 41 + 30	137 + 71 + 41 + 30
